# Poor Adherence to Self-Applied Topical Drug Treatment Is a Common Source of Low Lesion Clearance in Patients with Actinic Keratosis—A Cross-Sectional Study

**DOI:** 10.3390/jcm12113813

**Published:** 2023-06-01

**Authors:** Elias A. T. Koch, Theresa Steeb, Sophia Bender-Säbelkampf, Dorothee Busch, Janina Feustel, Matthias D. Kaufmann, Andreas Maronna, Christine Meder, Moritz Ronicke, Frédéric Toussaint, Hedwig Wellein, Carola Berking, Markus V. Heppt

**Affiliations:** 1 Department of Dermatology, Uniklinikum Erlangen, Friedrich-Alexander-University Erlangen-Nürnberg (FAU), 91054 Erlangen, Germany; elias.koch@uk-erlangen.de (E.A.T.K.); theresa.steeb@uk-erlangen.de (T.S.); hedwig.wellein@uk-erlangen.de (H.W.); carola.berking@uk-erlangen.de (C.B.); 2 Comprehensive Cancer Center Erlangen-European Metropolitan Area of Nürnberg (CCC ER-EMN), 91054 Erlangen, Germany

**Keywords:** actinic keratosis, topical treatment, adherence, compliance, consultation

## Abstract

Background: Many treatments for actinic keratosis (AK) have been proven efficient in clinical trials. However, patients with AK may still experience unsatisfactory therapeutic outcomes in clinical practice. Objectives: To investigate patient adherence to self-applied topical interventions for AK and to explore factors associated with adherence in a real-world setting. Methods: A cross-sectional study was conducted. Patients presenting with AK were asked to complete a self-administered questionnaire about their last topical AK treatment. Results: A total of 113 patients participated with a median age of 78.5 years (range 58–94). Fifty-four patients (47.8%) received topical diclofenac, ten (8.8%) imiquimod, nine (8%) 5-fluorouracil, nine (8%) 5-fluorouracil plus salicylic acid, and eight (7.1%) photodynamic therapy. The non-adherence rate was 46.9% (*n* = 53), and only 30.9% (*n* = 35) used the topical treatments according to the summary of product characteristics (SmPC). These subgroups were compared. Patients of the non-compliant group were significantly less informed about the application time of the specific topical intervention (*p* = 0.002) and adjusted the timeframe (*p* < 0.001) and application frequency of the therapy (*p* = 0.02) independently of their physician. Conversely, patients reporting a sufficient pre-treatment consultation (*p* = 0.019) generally complied with the SmPC compliance application. Conclusions: A thorough pre-treatment consultation can help to increase treatment adherence and ensure lesion clearance.

## 1. Introduction

Actinic keratoses (AKs) are common lesions of fair-skinned individuals occurring preferentially in the elderly with significant chronic sun exposure. They are among the most common conditions for which patients consult a dermatologist [1]. Single AK lesions bear a rather low risk of progression to invasive cutaneous squamous cell carcinoma (cSCC). However, the rate of progression increases rapidly with the presence of multiple AKs and additional clinical risk factors such as chronic immunosuppression or outdoor occupation [2,3]. Thus, guidelines consistently recommend treating AK as it is currently not possible to predict which AKs will transform into cSCC and when [4].

Treatment objectives are the clearance of clinical and subclinical lesions, the reduction in lesion relapse, the prevention of transformation into cSCC, and improvement in the aesthetic appearance. Numerous treatment approaches exist, ranging from ablative interventions to topical drug-mediated approaches [5,6]. The latter group is commonly prescribed by both dermatologists and general practitioners due to the ease of application and cost reimbursement by health insurance funds.

Commonly applied topical-drug-mediated interventions include 5-fluorouracil (5-FU), imiquimod (IMQ), diclofenac (DICLO), tirbanibulin (TIRBA), and photodynamic therapy (PDT). DICLO is a nonsteroidal anti-inflammatory drug (NSAID) from the phenylacetic acid class that has anti-inflammatory, analgesic, and antipyretic properties. It is available as a gel in a concentration of 30 mg/g in combination with hyaluronic acid 2.5% for the treatment of non-hyperkeratotic AK. The mechanism of action of DICLO is not entirely clear [5]. NSAIDs inhibit cyclooxygenase (COX) 1 and 2, enzymes that take part in the catalyzation of arachidonic acid to prostaglandins, and consequently the inflammatory prostaglandin cascade. The antitumoral effect of DICLO is based on the inhibition of COX-2 (which is overexpressed in AK and cSCC) and the inhibition of the prostaglandin E2 (associated with tumor angiogenesis and cell proliferation) [5,7,8]. Furthermore, DICLO can act as a partial agonist of the nuclear receptor PPARγ, leading to inhibition of cancer cell growth [9,10]. 5-FU is a cytostatic agent and an analog of the naturally occurring uracil (pyrimidine derivate), which is known as one of the four nucleobases in ribonucleic acid. 5-FU is approved as a monotherapy for the treatment of AK and can also be combined with salicylic acid 10%. Salicylic acid has keratolytic properties and improves the penetration of 5-FU in hyperkeratotic lesions [11]. The active pharmaceutical component is 5-fluorodeoxyuridine monophosphate, which inhibits the enzyme thymidylate synthase, which catalysis the conversion of deoxyuridine nucleotides to thymidine nucleotides. This inhibition leads to insufficient DNA and RNA production and induces apoptosis specifically in malignant cells due to faster DNA production [5,12]. IMQ, an analog of guanosine, is a synthetic compound that acts as an immune response modifier [5]. Initially, IMQ received approval for the topical treatment of external genital and perianal warts caused by the human papillomavirus, due to its potent antiviral activity. During clinical investigations, IMQ also demonstrated efficacy in the treatment of skin lesions induced by ultraviolet light [13,14]. The mechanism of action of IMQ involves the activation of Toll-like receptor-7 (TLR7) [15]. This activation leads to the synthesis and release of IFN-α, IFN-γ, TNF-α, interleukins (IL-1, IL-6, IL-12), GM-CSF, and other cytokines from dermal macrophages, monocytes, and epidermal keratinocytes [16]. Consequently, IMQ enhances both the nonspecific innate immune response and the cellular arm of the acquired immune response. This cascade results in the infiltration of immune cells and increases local cytokine production at the site of application with increased antiviral and antitumor activities [5,17,18]. TIRBA is a small-molecule inhibitor belonging to the class of peptidomimetics. TIRBA selectively and highly specifically inhibits microtubule polymerization, which induces p53 expression, arrest of cell division in interphase gap 2, and subsequent apoptosis by stimulating caspase-3 and cleavage of poly(adenosine diphosphate (ADP)-ribose) polymerase [19]. Due to this specific inhibition, TIRBA shows a good tolerability profile [20]. PDT is a primarily field-directed approach based on the activation of photosensitizing agents applied to the skin surface. Typically, aminolevulinic acid or its ester methyl-5-aminolevulinate is used, leading to the production and accumulation of proto-porphyrins in precancerous keratinocytes. When illuminated by the light of an appropriate wavelength either by light-emitting diodes (LEDs) or daylight, a phototoxic reaction is triggered. This causes the irreversible oxidation of cellular components and consequent apoptosis of the target cells and necrosis [5,21].

All these therapies have been proven efficient in pivotal clinical trials [17,20,22,23,24]. Nevertheless, in daily clinical practice, the clearance rates are often lower than in randomized controlled trials (RCTs). Treatment adherence is generally higher in RCTs than in the real-world setting. Non-compliance may represent an important source of treatment resistance and poor lesion clearance, but data on the correct application of topical drugs outside of RCTs are sparse [14,25,26,27]. A study in the UK examined the adherence to topical treatments for AK, revealing a high rate of non-adherence (63%) and non-persistence to the desired treatment duration (31%) [28].

Here, we present the results of a cross-sectional study that aimed to investigate patient adherence to self-applied topical interventions for AK in the setting of a large university hospital. We explore factors that were associated with poor or non-adherence to treatment and derive solutions to improve treatment outcomes.

## 2. Materials and Methods

### 2.1. Setting and Study Population

A questionnaire-based study including patients with a clinical diagnosis of AK presenting to the Department of Dermatology at Uniklinikum Erlangen, Germany, was conducted from April 2020 to 2021. Patients were selected via purposive sampling, i.e., the physician chose the best-fit participants for this survey according to availability. Only patients who had undergone a self-applied topical intervention for their AK within the previous 12 months were included. The participants were asked to complete a standardized questionnaire alone or, if needed, with assistance from a physician. The questionnaire was reviewed and approved by an independent research ethics committee of Friedrich-Alexander-University Erlangen-Nürnberg (approval number 125_20 Bc). Anonymous participation was voluntary, and each participant was only allowed to participate once in the survey (cross-sectional design). The data collection was anonymized. No incentives were provided.

### 2.2. Description of the Questionnaire

As no validated survey tools for the objective of our study existed, the questionnaire was developed de novo based on a literature review and dermato-oncological expert consultation. It included questions on disease-specific information such as localization and number of AKs as well as treatment-related information such as the topical treatment agent, frequency, and duration of application.

Further questions addressed information about the pre-treatment consultation and the self-estimated clearance rate for the topical treatment. The preferred mode of application was assessed with additional pictures for the sake of visual illustration (e.g., tube, sachet, pump dispenser, roll-on, brush applicator). In another series of questions, a visual analog scale (VAS) ranging from 0 to 10 (0 = strongly disagree, 10 = strongly agree) was used to investigate items concerning pre-treatment consultation, application, and side effects. Solutions to improve the treatment and adherence were presented, which the patient could rate with the same scale from 0 to 10. In addition, sociodemographic characteristics were recorded (sex, age, marital status, highest level of education, and insurance status). Participants could also add further comments and suggestions for improvement of therapy in a free-text field. The full questionnaire is available upon request from the authors. The survey was pre-tested and validated by independent researchers and patients for clarity and comprehension and revised to the final form based on their suggestions. Completed questionnaires were not linked to any identifying patient information to ensure irreversible anonymity.

### 2.3. Subgroup Selection

The treatment-related information along with the summary of product characteristics (SmPC) of the specific topical drug was used to inform whether the application was SmPC-compliant or not (SmPC-non-compliant). This method was selected due to its small bias compared to the questions if they had followed their physician’s recommendation as a proxy for non-adherence. Furthermore, we compared patients that indicated a clearance rate of their AK of less than 50% with those who indicated a higher healing rate as a proxy for the treatment efficacy.

### 2.4. Statistical Analysis

All patients who completed the questionnaire were included in the analysis. All analyses were carried out with the software IBM SPSS Statistics 28 version (IBM, Armonk, NY, USA). Descriptive statistics were used to describe patient demographics, and the results are reported as frequencies and their percentage (%) including standard deviation (SD). Free-text fields were categorized into subgroups based on patient recommendations and expressed as a percentage. Rank correlation models (Pearson) and chi^2^ tests were used to test associations between two variables. Student t-tests were performed to evaluate differences between diverse groups. A *p*-value < 0.05 was considered statistically significant, and a value <0.01 was considered significant for the rank correlation model (Pearson). Missing data were not imputed.

## 3. Results

**Figure 1:**
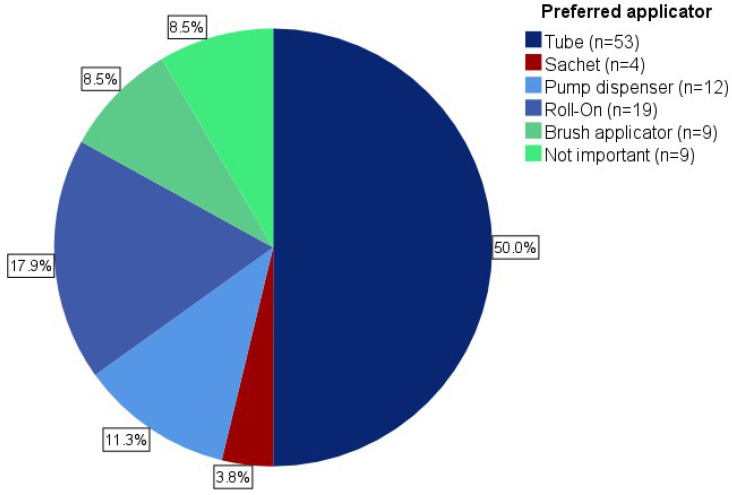
Preferred routes of application.

Overall, 113 questionnaires were returned; 16 patients were female (14.2%), 95 were male (84.1%), and 2 did not specify (1.8%; Table 1). The median age was 78.5 years (range 58–94); 84 patients (74.3%) had statutory health insurance, 26 private (23%), and 3 (2.7%) did not provide information. In the previous 12 months, 54 patients (47.8%) received therapy with DICLO, 10 (8.8%) with IMQ, 9 (8%) with 5-FU, 9 (8%) with 5-FU 0.5% and salicylic acid 10%, and 8 (7.1%) with PDT. Nine patients (8%) reported more than one treatment regimen and fourteen (12.4%) did not further specify. Forty-six participants (40.7%) had 1-5 AK lesions, thirty-five had (31%) 5–10, and twenty-two had (19.5%) > 10, which were most commonly located on the scalp (*n* = 40, 43.4%). Sixty-four patients (56.6%) went to see the prescribing physician for treatment follow-up. Of these, 73.4% (*n* = 47) presented later than 2 months, 21.9% (*n* = 14) within the first 2 months, and 3.1% (*n* = 2) within the first 2 weeks after treatment initiation. The duration of the pre-treatment consultation was reported as 2–5 min in 46.9% of cases (*n* = 53), more than 5 min in 28.3% (*n* = 32), and less than 2 min in 19.5% (*n* = 22). Notably, 50% (*n* = 53) of patients preferred the classic tube as an application form, whereas 17.9% (*n* = 19) voted for the roll-on, 11.3% (*n* = 12) for the pump dispenser, and 8.5% (*n* = 9) for the brush applicator (Figure 1).

Patients indicated a mean VAS score of 6.5 (*n* = 106, SD = 2.8) for how sufficiently informed about AK therapy they felt, and a mean VAS score of 5.76 (*n* = 108, SD = 3.3) for how informed they felt about side effects. Overall, the perception of strong side effects was low (VAS 2.7, *n* = 97, SD = 3.1); patients were not scared of side effects (VAS 2.1, *n* = 100, SD = 2.7) and the therapy was rarely discontinued due to side effects (VAS 2.1, *n* = 85, SD = 3.2). Participants rated whether they felt that had received family support with a mean VAS score of 4.6 (*n* = 100, SD = 4.0) (Table 2).

**Figure 2:**
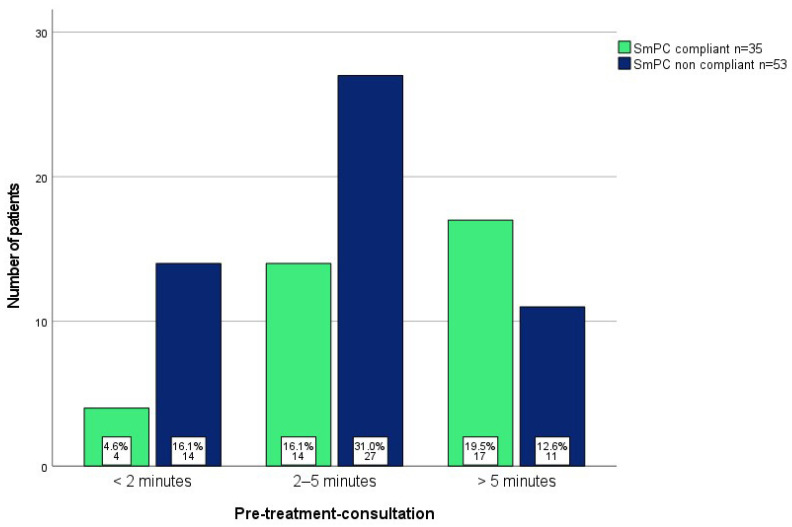
Bar diagram illustrating the number of patients who applied the topical intervention for AK according to the Summary of Product Characteristics (SmPC-compliant) or not (SmPC-non-compliant), stratified by the length of the pre-treatment consultation (presented in percentages and number of patients). Patients with a longer consultation showed significantly less SmPC-non-compliant application (Pearson: *p* = 0.006, correlation coefficient = 0.293, *n* = 87, chi^2^: *p* = 0.019).

Based on the information provided in the questionnaire, 53 patients (46.9%) used the prescribed topical drugs according to the SmPC (SmPC-compliant), while 35 (30.9%) did not (SmPC-non-compliant). Notably, patients with SmPC-non-compliant application were significantly less informed about the application duration (*p* = 0.02) and adjusted the duration of therapy without consulting their physician (*p* < 0.001). There were no differences in their perceptions of side effects, fear of side effects, or discontinuation of treatment due to side effects. Further details on these subgroup analyses are listed in Table 2. Furthermore, SmPC-non-compliant application was correlated with sociodemographic data and questions concerning medical consultation. Patients with a longer pre-treatment consultation (Pearson: *p* = 0.006, correlation coefficient = 0.293; chi^2^ test: *p* = 0.019) showed significantly less non-compliant application (Figure 2, Supplementary Table S1).

Forty-four patients (38.9%) indicated an AK clearance rate of less than 50%, and fifty-six patients (49.6%) had a higher clearance rate as a proxy for the treatment efficacy. Thirteen participants did not specify the clearance rate. Patients who stated a clearance of <50% were significantly less informed about application duration and the duration of therapy (*p* = 0.05, *p* = 0.04, respectively). No differences in side effects or family support were evident (Supplementary Table S2).

Twenty-five patients (22.1%) used the free-text field to share their ideas for the improvement of therapy, 72% (18/25) wished they had had a sufficient pre-treatment consultation with the physician, and 36% (9/25) suggested further assistance in the treatment application was needed. Furthermore, 44.4% (4/9) suggested a visual representation of the application should be provided, while 55.6% (5/9) of patients wished they had had both an improved pre-treatment consultation and further assistance. Two patients (8%, 2/25) suggested a demonstration by the physician, and another patient (4%, 1/25) recommended studying the SmPC (Supplementary Table S3).

## 4. Discussion

AKs are very common skin lesions generally caused by chronic sun damage. Several treatment options are available for the treatment of AKs, which makes the selection of treatment challenging for physicians. Furthermore, while the different endpoints used in studies restrict their comparability, the different topical treatments, such as 5-FU, IMQ, and DICLO, have all proven to be efficient in clinical trials [14,26,29]. However, in daily practice, some patients may experience lower AK clearance and higher recurrence rates than in RCTs. Treatment adherence is generally higher in clinical trials than in clinical practice outside of trials [27]. We found in our study population a high non-adherence rate of 46.9% (*n* = 53), and only 30.9% (*n* = 35) used the drug according to the SmPC, which might be the reason why poorer clearance rates were observed in this study than in pivotal trials. These results are in line with a questionnaire-based study from the UK, which found that 63% of patients were non-adherent to their topical therapy protocol for AK, and as many as 88% of patients were either non-adherent, non-persistent, or both [28]. In a another longitudinal diary study conducted in Germany, France, and the UK, 75% of patients adhered to the prescribed interval dosing regimen, but only 36% adhered to the treatment duration [30].

Our analysis goes beyond these examinations: We reveal that patients with SmPC-non-compliant application were significantly less informed about the application time (leave on time) of the specific topical drug (*p* = 0.002) and adjusted the duration (*p* < 0.001) and dosing frequency of the therapy (*p* = 0.02) independently of their physician’s instructions. Nevertheless, the patients rated the level of information they felt they had received about the topical drug application as 6.5/10 on the VAS. Additionally, patients reporting a long pre-treatment consultation generally complied with the correct treatment regimen. Furthermore, patients with low clearance rates were significantly less informed about application times and the duration of the treatment, suggesting that there is a connection between pre-treatment consultation, SmPC-compliant application, and treatment success. These data provide evidence that the pre-treatment consultation needs to be taken seriously by the physician to ensure ideal clinical outcomes. Notably, patients rated the items “occurrence of AE” (2.7/10), “fear of AE” (2.1/10), and “discontinuation of therapy due to AE” (2.1/10) rather low, with no differences between the groups of SmPC-compliant and non-compliant application. These results suggest that the domain of AE did not impact treatment adherence in this population, which was not assessed in a previous longitudinal diary study [31]. In a more recent cross-sectional questionnaire-based study, only a few patients feared AE in the treatment of AK (6.8%), which is consistent with this study. Concerns were mainly related to the duration of therapy (30%) and its effectivity (24.5%) [32].

In addition, in our survey, socioeconomic factors such as a higher education level were not associated with SmPC-compliant application. Others reported that patients of a lower socioeconomic status were more prone to non-adherence and discontinuation of oral cancer therapy [33,34]. In breast cancer patients, a systematic review examined the relationship of psychosocial factors with their adherence to oral anti-cancer medication and found that a good patient–physician relationship is crucial for adherence to treatment regimens [35]. Non-adherence to oral anti-cancer medication was associated with a lower survival rate and higher risk of recurrence [36]. Furthermore, the patient’s perception of a physician’s empathic skills and competence correlated with expected treatment outcomes, which in turn correlated with adherence [37,38].

Considering the often-hectic clinical routine in dermatological outpatient clinics, there is usually little time for detailed education of patients undergoing topical drug treatment. Nevertheless, our data provide evidence that sufficient pre-treatment consultation is important to ensure treatment adherence and, ultimately, successful AK clearance. Thus, patients with several AKs and especially patients with field cancerization should be referred to specialized consultation hours, or supporting interventions should be offered. Most of the patients in our survey disagreed that they would have liked supporting interventions to improve adherence (see Table 2), whereas 36% of patients using the free-text field stated that they wished they had had further assistance and supporting interventions, such as a written patient information brochure including simple language, no technical terms, short sentences, and pictures. Especially considering the median age of the study population of 78.5 years, the associated decline in cognitive abilities implies the importance of simple supporting interventions. Interventions including patient education, written action plans, and a quick return for a follow-up visit have been proven to increase adherence [39]. However, in increasingly globalized and digitalized societies, more modern methods, such as applications (“App”) for mobile phones with important information, electronic reminders, and training videos, will become increasingly relevant in the future. However, the implications of such techniques present a challenge in particular among the elderly, and an initiative to strengthen patient orientation and patient information has recently been launched by the Skin Cancer Council (NVKH) in Germany [40].

The limitations of this survey include the performance of this study as a single-center design, although many patients received therapy from their practitioners. As the patients had to complete the questionnaire based on their recollection of previous topical treatments, recall bias cannot be excluded. Furthermore, the sample size of this study was relatively small, and it was not sampled in a random fashion. The recruitment process depended on the availability of patients, which might imply a sampling bias. Furthermore, we recorded the patients’ self-reports, which may result in an improved self-representation [28,41]. However, we tried to limit this bias by asking patients to describe how frequently and for how long they used the drug without any reference to the SmPC. Moreover, information on how a topical substance should be applied does not necessarily equate to health-related competence of the patient. The perception and processing of the information given in a pre-treatment consultation also depend on other factors, such as cultural influences, the personal level of education, and the subjective attention of the patient during the consultation. Therefore, the suggestion of a sole reduction in the length of the pre-treatment consultation may be an overly simplified solution. In addition, some topical substances are inherently more complicated to explain than others, which could further bias the results.

## 5. Conclusions

This study provides evidence that insufficient pre-treatment consultation is associated with SmPC-non-compliant application and suggests that longer and better instruction on the use of topical drugs can increase their correct use, adherence to treatment, and AK clearance. Patients could be referred to specialized consultation hours, or supporting interventions could be applied. The preferred application form is still the classic tube.

## Figures and Tables

**Table 1:** Baseline characteristics of the study population. Abbreviations: AK = actinic keratosis, SmPC = summary of product characteristics.

Demographics	Median (Range)		*n*	%
Age		78.5 (58–94)	110	97.3
	Missing		3	2.7
Gender	Male		95	84.1
	Female		16	14.2
	Missing		2	1.8
Marital status	Single		5	4.4
	Married		82	72.6
	Divorced		5	4.4
	Widowed		19	16.8
	Missing		2	1.8
Insurance	Statutory		84	74.3
	Private		26	23
	Missing		3	2.7
Education	No degree		1	0.9
	Secondary school (lower)		42	37.2
	Secondary school (higher)		20	17.7
	High school		8	7.1
	University/college		38	33.6
	Missing		4	3.5
Topical drug	5-Fluoruracil 0.5% and salicylic acid 10% solution		9	8
	Imiquimod cream 5% or 3.75%		10	8.8
	Diclofenac natrium gel 3% in hyaluronic acid 2.5% gel		54	47.8
	5-Fluoruracil cream 5%		9	8
	Photodynamic therapy		8	7.1
	>1 treatment		9	8
	Missing		14	12.4
Number of AKs	1–5 AKs		46	40.7
	5–10 AKs		35	31
	>10 AKs		22	19.5
	Missing		10	8.8
Localization	Scalp		49	43.4
	Trunk		2	1.8
	Face		26	23
	Extremities		8	7.1
	>1 incl. head		24	21.2
	>1 excl. head		4	3.5
	Missing		0	0
Drug use	SmPc-compliant		53	31
	SmPC-non-compliant		35	46.9
	Missing		25	22.1
Duration of pre-treatment consultation	<2 min		22	19.5
	2–5 min		53	46.9
	>5 min		32	28.3
	Missing		6	5.3
Consultation in the treatment follow-up	No consultation		42	37.2
	Consultation		64	56.6
	Missing		7	6.2
Time interval of consultation in the treatment follow-up	<2 weeks		2	3.13
	1–2 months		14	21.88
	>2 months		47	73.44
	Missing		1	1.55

**Table 2:** Ratings for the domains “drug application” and “improvement of treatment education” in the overall population and the subgroups with SmPC-compliant and SmPC-non-compliant application. Ratings were performed on a visual analog scale from 0 to 10.

Questions (0–10 Scale; 0 = Strongly Disagree, 10 = Strongly Agree)		All (*n* = 113) Mean (*n*) Standard Deviation = SD	Patients with SmPC-Compliant Application (*n* = 35) Mean (*n*) Standard Deviation = SD	Patients with Smpc Non-Compliant Application (*N* = 53) Mean (*n*) Standard Deviation = SD	*p*-Value (*t*-Test)
Domain “drug application”	felt sufficiently informed	6.5 (106) SD = 2.82 Missing = 7	6.97 (35) SD = 2.61	6.11 (50) SD = 3.15	0.19
	informed about side effects	5.76 (108) SD = 3.31 Missing = 5	6.34 (35) SD = 2.92	5.2 (50) SD = 3.78	0.14
	informed about frequency of application	7.32 (99) SD = 2.62 Missing = 14	7.14 (29) SD = 2.33	7.5 (50) SD = 2.75	0.54
	informed about the maximal area that could be treated	6.73 (100) SD = 2.95 Missing = 13	6.76 (29) SD = 2.65	6.87 (50) SD = 3.05	0.86
	informed about application time	6.56 (101) SD = 2.97 Missing = 12	7.42 (30) SD = 2.38	5.91 (50) SD = 3.32	0.02
	informed about duration of therapy	6.56 (102) SD = 2.91 Missing = 11	7.18 (31) SD = 2.3	6.15 (50) SD = 3.36	0.14
	adjusted duration independently of prescribing physician	4.5 (97) SD = 3.56 Missing = 16	2.59 (29) SD = 3.05	5.53 (50) SD = 3.53	<0.001
	adjusted frequency independently of prescribing physician	4.27 (97) SD = 3.5 Missing = 16	3.16 (29) SD = 3.31	5.16 (50) SD = 3.58	0.02
	received family support for correct application	4.62 (100) SD = 3.95 Missing = 13	4.72 (30) SD = 3.5	4.93 (50) SD = 4.23	0.81
	experienced strong side effects	2.72 (97) SD = 3.12 Missing = 16	2.35 (29) SD = 2.6	2.67 (50) SD = 3.21	0.64
	scared of side effects	2.1 (100) SD = 2.65 Missing = 13	1.97 (30) SD = 2	2.14 (50) SD = 2.78	0.76
	discontinuation of treatment due to side effects	2.07 (85) SD = 3.23 Missing = 28	0.96 (24) SD = 2.19	2.35 (44) SD = 3.5	0.08
Domain “improvement of treatment education”	training before therapy	3.77 (104) SD = 3.49 Missing = 9	2.94 (33) SD = 3.26	4.3 (52) SD = 3.51	0.06
	training video	3.67 (102) SD = 3.6 Missing = 11	3.47 (31) SD = 3.3	3.72 (51) SD = 3.7	0.78
	reminder application (APP, mobile phone)	1.97 (102) SD = 2.88 Missing = 11	1.69 (32) SD = 2.47	2.27 (51) SD = 3.14	0.37
	treatment diary	2.12 (102) SD = 2.79 Missing = 11	2.31 (31) SD = 3.28	1.96 (52) SD = 2.36	0.58
	application information online	2.32 (102) SD = 2.97 Missing = 11	2.3 (32) SD = 3.26	2.25 (51) SD = 2.85	0.91
	written application information	4.75 (103) SD = 3.7 Missing = 10	4.14 (33) SD = 3.6	4.9 (50) SD = 3.83	0.38

## Data Availability

All questionnaires and study-related data are stored at the Universitätsklinikum Erlangen, Friedrich-Alexander-University Erlangen-Nürnberg (FAU), 91054 Erlangen, Germany, and are available upon reasonable request.

## Supplementary Materials

The following supporting information can be downloaded at: https://www.mdpi.com/article/10.3390/jcm12113813/s1. Table S1: Cross-table presenting SmPC (non) compliant application and duration of pre-treatment-consultation (Chi² test, p = 0.019). Table S2: Ratings for the domains “drug application” and “improvement of treatment education” in the overall population and the subgroups with ≥50% and <50% cleared lesions. Ratings were performed on a visual analog scale from 0 to 10. Table S3: Summary of the 25 patients (22.1%) using the free-text field to share their ideas for improvement of treatment education.
