# Supplementary material for: Poor Adherence to Self-Applied Topical Drug Treatment Is a Common Source of Low Lesion Clearance in Patients with Actinic Keratosis—A Cross-Sectional Study
Source: J Clin Med. 2023 Jun 1;12(11):3813. doi: 10.3390/jcm12113813 (PMC10253365; doi:10.3390/jcm12113813)
Supplement: Supplementary file 1 [file jcm-12-03813-s001.zip › jcm-2407007-supplementary.pdf]

Supplementary Table S1. Cross-table presenting SmPC (non) compliant application and duration of pre-treatment-consultation (Chi² test, p=0.019).

			SmPC compliant	SmPC non-compliant
**Pre-treatment consultation**	**< 2 minutes**	Number	4	14
		Expected number	7.2	10.8
	2-5 minutes	Number	14	27
		Expected number	16.5	24.5
	> 5 minutes	Number	17	11
		Expected number	11.3	16.7

Supplementary Table S2. Ratings for the domains "drug application" and "improvement of treatment education" in the overall population and the subgroups with ≥50% and <50% cleared lesions. Ratings were performed on a visual analog scale from 0 to 10.

**Questions (0 - 10 scale; 0 = strongly disagree, 10 = strongly agree)**		All (n=113) mean (n)	>=50% healed AK (n=56) mean (n)	<50% healed AK (n=44) mean (n)	p-value (T-Test)
**Domain "drug application"**	felt sufficiently informed	6.5 (106) SD = 2.82 Missing = 7	6.7 (55) SD = 2.83	5.98 (42) SD = 2.79	0.16
	informed about side effects	5.76 (108) SD = 3.31 Missing = 5	5.9 (55) SD = 3.36	5.16 (44) SD = 3.38	0.28
	informed about frequency of application	7.32 (99) SD = 2.62 Missing = 14	7.74 (52) SD = 2.17	6.83 (41) SD = 2.92	0.09
	informed about maximal area to treat	6.73 (100) SD = 2.95 Missing = 13	6.71 (54) SD = 2.99	6.9 (40) SD = 2.83	0.72
	informed about application time	6.56 (101) SD = 2.97 Missing = 12	6.98 (54) SD = 2.9	5.83 (41) SD = 3.01	0.05
	informed about duration of treatment	6.56 (102) SD = 2.91 Missing = 11	7.02 (53) SD = 2.73	5.76 (42) SD = 3.09	0.04
	adjusted duration independently of prescribing physician	4.5 (97) SD = 3.56 Missing = 16	4.53 (53) SD = 3.68	4.74 (38) SD = 3.37	0.76
	adjusted frequency independently of prescribing physician	4.27 (97) SD = 3.5 Missing = 16	4.08 (53) SD = 3.46	4.66 (38) SD = 3.48	0.44
	Received family support for correct application	4.62 (100) SD = 3.95 Missing = 13	4.8 (54) SD = 3.97	3.89 (39) SD = 3.91	0.27
	Experience of strong side effects	2.72 (97) SD = 3.12 Missing = 16	2.93 (54) SD = 3.24	2.36 (39) SD = 3.05	0.41
	Scared of side effects	2.1 (100) SD = 2.65	2.06 (55) SD = 2.74	2.04 (39) SD = 2.46	0.94

		Missing = 13			
	Discontinuation of treatment due to side effects	2.07 (85)<br>SD = 3.23<br>Missing = 28	1.87 (45)<br>SD = 2.98	2.25 (36)<br>SD = 3.4	0.58
**Domain “improvement of treatment education”**	Training before therapy	3.77 (104)<br>SD = 3.49<br>Missing = 9	3.85 (55)<br>SD = 3.51	4.04 (42)<br>SD = 3.41	0.78
	Training video	3.67 (102)<br>SD = 3.6<br>Missing = 11	4.04 (55)<br>SD = 3.77	3.58 (40)<br>SD = 3.41	0.53
	Reminder application (APP, mobile phone)	1.97 (102)<br>SD = 2.88<br>Missing = 11	1.85 (53)<br>SD = 2.88	2.43 (42)<br>SD = 3.03	0.33
	Treatment Diary	2.12 (102)<br>SD = 2.79<br>Missing = 11	1.96 (53)<br>SD = 2.7	2.22 (41)<br>SD = 2.7	0.65
	Application information online	2.32 (102)<br>SD = 2.97<br>Missing = 11	2.66 (53)<br>SD = 3.18	2.12 (42)<br>SD = 2.81	0.4
	Written Application information	4.75 (103)<br>SD = 3.7<br>Missing = 10	4.32 (54)<br>SD = 3.73	5.26 (42)<br>SD = 3.57	0.21

Supplementary Table S3. Summary of the 25 patients (22.1%) using the free-text field to share their ideas for improvement of treatment education.

Categories	Percentage	Quotes (representable)
Sufficient pre-treatment consultation by the prescribing physician	72% (18/25)	“Personal consultation. Concise guide like IKEA furniture, if necessary a learning video with max. 3 minutes. Important: simple language, no technical terms, short sentences, supported by pictures.”
		“By providing comprehensive information about the therapy regardless of the medium.”
Further assistance in the treatment application, (5/9) patients wished for both, an improved pre-treatment consultation and further assistance	36% (9/25)	“On the basis of a picture, exact record of the places to be treated, possibly the indication where the treatment should not be performed under any circumstances.”
Demonstration by the physician	8% (2/25)	“The first time the physician puts cream on.”
		“Regular dermatological checks, demonstration of application by dermatologist.”
Studying the SmPC	4% (1/25)	-
